# Functional features of a novel interferon-stimulated gene SHFL: a comprehensive review

**DOI:** 10.3389/fmicb.2023.1323231

**Published:** 2023-12-11

**Authors:** Xingzheng Wang, A-Mei Zhang

**Affiliations:** Faculty of Life Science and Technology, Kunming University of Science and Technology, Kunming, Yunnan, China

**Keywords:** interferon-stimulated genes, SHFL, antiviral effects, RNA viruses, ribosomal frameshifting

## Abstract

Various interferon (IFN)-stimulated genes (ISGs), expressed via Janus kinase–signal transducer and activator of transcription (JAK-STAT) signaling pathway-stimulated IFNs to increase antiviral effects or regulate immune response, perform different roles in virus-infected cells. In recent years, a novel ISG, *SHFL*, which is located in the genomic region 19p13.2 and comprises two isoforms, has been studied as a virus-inhibiting agent. Studies have shown that SHFL suppressive effects on human immunodeficiency virus-1 (HIV), Zika virus (ZIKV), dengue virus (DENV), hepatitis C virus (HCV), Japanese encephalitis virus (JEV), porcine epidemic diarrhea virus (PEDV), Human enterovirus A71 (EV-A71) and Kaposi’s sarcoma-associated herpes virus (KSHV). SHFL interacts with various viral and host molecules to inhibit viral life circle and activities, such as replication, translation, and ribosomal frameshifting, or regulates host pathways to degrade viral proteins. In this review, we summarized the functional features of SHFL to provide insights to underlying mechanisms of the antiviral effects of SHFL and explored its potential function.

## 1 Introduction

In the world, human beings and other animals are affected by various viruses. Organisms exposed to virus could still maintain healthy condition through the self-defense system of the host, which monitors and provides antiviral ability against pathogens and further activates immune responses of host (Skariah et al., 2022). Interferons (IFNs) were first discovered in 1957 by Isaacs and Lindenmann (1988). Then, three IFN families, including type I (α), II (β), and III (γ) IFNs, have been known to play an effective role in cellular self-defense, forming a robust first-line innate defense of host. Although IFNγ has no notable reaction to most immune cells, IFNα and IFNβ are pivotal for antiviral activities (Durbin et al., 2013). Regardless, intracellular signaling cascades can be induced by all three types of IFN via Janus kinase–signal transducer and activator of transcription (JAK-STAT) pathway, triggering the expression of IFN-stimulated genes (ISGs) that exert multiplex biological and pathological functions (Chen et al., 2017).

**Figure 1:**
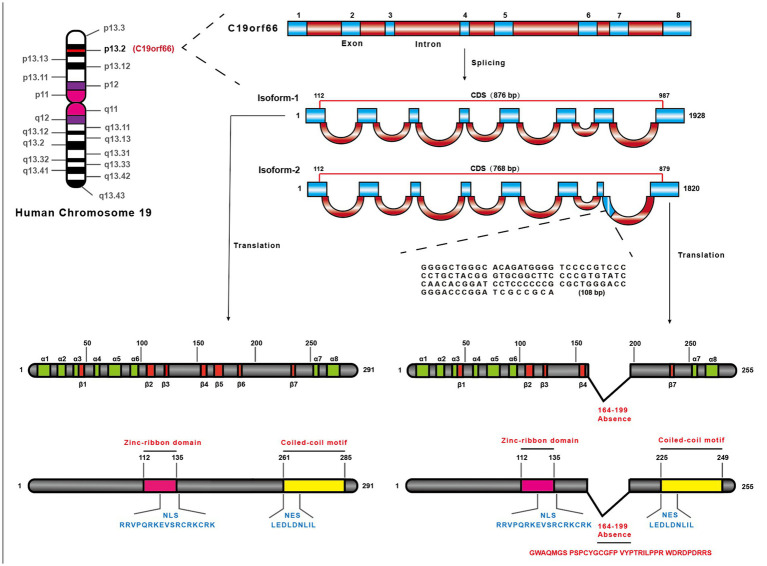
The location of SHFL on the human chromosome and structural characteristics. SHFL is situated on Human chromosome 19 the region p13.2, containing eight exons, and produces two isoforms caused by the alternative splicing. And the shorter one (768 bp) (NM_001308277.2), isoform-2, is different from isoform-1 (876 bp) (NM_018381.4) in the absence of 164–199 nucleotides on the seventh exon. Isoform-1 as the longer one, encoding 291 amino acids, also possesses some distinctions compared with isoform-2 which only encodes 255 amino acids. The prediction of the protein structure of isoform-1 of SHFL exposits that the isoform-1 consists of eight-α-helices and seven β-strands, and possesses the nuclear export signal (261–269 amino acids), nuclear localization signal (121–137 amino acids), a zinc-ribbon domain (112–135 amino acids), and a coiled-coil motif in C-terminus (261–285 amino acids). And the lack region of isoform-2 constitutes part of the fifth β-strand and the entire sixth β-strand.

The shiftless antiviral inhibitor of ribosomal frameshifting (*SHFL*) gene was firstly predicted as an ISG in 2011 (Schoggins et al., 2011), which is also named as C19orf66, FLJ11286, IRAV, UPF0515, and RyDEN. Two isoforms were constructed by eight exons of the *SHFL* gene. The longer transcript (876 nt, NM_018381.4) encodes 291 amino acids (isoform-1), and the shorter one (768 nt, NM_001308277.2) encodes 255 amino acids (isoform-2) that lacks 164th–199th amino acid compared with isoform-1. The protein structure prediction of isoform-1 of SHFL revealed eight α-helices, seven β-strands, a zinc-ribbon domain (112th–135th amino acid), a nuclear localization signal (NLS, 121st–137th amino acid), a nuclear export signal (261st–269th amino acid), and a coiled-coil motif at the C-terminus (261st–285th amino acid) (Figure 1; Suzuki et al., 2016). SHFL showed high expressing level in human liver and spleen tissues, whereas a lower expression is observed in the pancreas and heart.^1^ Because the liver and spleen are important for human immune defense and metabolism, the high expression of SHFL in these tissues may be associated with those functions.

SHFL was then detected with higher expressing level in cells infected by many viruses, such as immunodeficiency virus (HIV)-1, Zika virus (ZIKV), dengue virus (DENV), HCV, Japanese encephalitis virus (JEV), porcine epidemic diarrhea virus (PEDV), and Kaposi’s sarcoma-associated herpesvirus (KSHV), and was reported as a broad-spectrum virus-inhibiting ISG (Rodriguez and Muller, 2022). Because a protein structure could determine its function, the zinc-finger and coiled-coil domains of SHFL might be dominant to its diverse antiviral functions. In this review, we summarized the functional properties of SHFL and its underlying antiviral mechanisms against viruses with distinctively-characterized genomes to provide a theoretical basis for systematically understanding this gene.

## 2 The inhibiting function of SHFL to RNA viruses

Many viruses belong to RNA virus family, whose genomes are positive or negative RNA (Villa et al., 2020). However, SHFL seems to indulge in the viruses of the *Flaviviridae* family. SHFL plays important antiviral role in DENV, ZIKV, HCV, and JEV infection. Additionally, HIV, PEDV and EV-A71 also can be inhibited by SHFL (Rodriguez and Muller, 2022).

### 2.1 SHFL binds with complex of NS3 and NS4A to inhibit DENV

DENV is an enveloped, positive-sense, single-stranded RNA virus, covering four serotypes (DENV 1–4). DENV has promptly disseminated across many countries and regions worldwide, arousing dengue fever and dengue hemorrhagic fever in humans (Guzman and Harris, 2015). The RNA encodes three structural proteins including capsid (C), envelope (E) and membrane (M) and seven non-structural proteins, including NS1, NS2A, NS2B, NS3, NS4A, NS4B and NS5 (Kok et al., 2023).

SHFL could be up-regulated by DENV infection, and it interacts with complex of NS3 and NS4A to inhibit DENV replication. Moreover, DENV RNA also could be band by SHFL during translation (Suzuki et al., 2016; Balinsky et al., 2017). Additionally, some host molecules can also be associated with SHFL, such as poly(A) binding protein cytoplasmic 1 (PABPC1), which can stimulate the DENV replication, along with the P body and MOV10, which are both related to the stress response.

These studies showed the main process and molecules that can be affected by SHFL and the negative effect on DENV. Interestingly, the mutation of NLS domain in the *SHFL* gene lead to a decreasing binding level with DENV (Suzuki et al., 2016). The arginine (R) and lysine (K) are most abundant amino acid in NLS domain (Boulikas, 1994). Thus, mutation of R and K might change the SHFL anti-DENV activity by destroyed structure. Since the NLS domain is important for SHFL functioning, we speculated that the zinc-finger domain probably also plays a crucial role in restricting DENV due to the 15 amino acids shared between the two domains.

### 2.2 Against ZIKV through down-regulating NS3 of ZIKV

ZIKV, a member of the *Flaviviridae* family, is a positive-sense, single-stranded RNA virus. Similar to DENV, its genomic RNA encodes three structural proteins and seven nonstructural proteins (Sirohi and Kuhn, 2017). Notably, the proteins NS2, NS3, and NS5 consists of RNA-dependent RNA polymerase (RdRp) and are necessary for ZIKV replication (White et al., 2016; Wu et al., 2020).

SHFL can restrain the expression of ZIKV RNA and the viral particles, and further prevent ZIKV replication. When interacting with NS2B and NS3 proteins of ZIKV, SHFL downregulates NS3 through the lysosome-dependent pathway. Although SHFL can also suppress the ZIKV envelope protein, the mechanism is still unknown (Wu et al., 2020). Interestingly, knocking out of SHFL in mice increased the ZIKV RNA expressing level, demonstrating that the mouse ortholog of SHFL (Shfl) restricts ZIKV *in vivo* (Hanners et al., 2021).

SHFL still inhibited viral growth via its interaction ability, but it also affected NS3 protein via the lysosome-dependent pathway. This indicates that SHFL exert anti- ZIKV activity together with the lysosome-dependent signal pathway. In the Hanners’ study (Hanners et al., 2021), the results of Shfl against ZIKV in mouse model suggest a possibility of the similarity in the pivotal structure of SHFL and its ortholog. Thus, SHFL might also play antiviral roles in other species due to its conservative structure.

### 2.3 Damage of HCV membrane web by SHFL

HCV is a positive-sense, single-stranded RNA flavivirus, and its genomic compositions are similar to ZIKV and DENV. The NS proteins of HCV regulate viral replication and other activities (Darius and François, 2013). Remarkably, HCV NS5A can induce phosphatidylinositol-4-phosphate (PI[4]P) production, which can promote the formation of the membranous webs and provide a platform for HCV replication (Bishé et al., 2012; Blanchard and Roingeard, 2018).

SHFL co-locates in the cellular compartment with HCV replicase complex, and it could inhibit HCV replication by decreasing the NS5A level, which is a key factor in HCV replication. Additionally, SHFL inhibits HCV by reconstructing membranous web. Mutations in zinc-finger domain of SHFL reduce the anti-HCV activity, and it means that the zinc-finger domain is a pivotal structure for SHFL in anti-HCV role in the infected cells (Kinast et al., 2020). This indicated that zinc-finger domain might play important role in SHFL location. Recently, Shfl was also reported to own the anti-HCV ability in mouse model (Zhang et al., 2023). These studies suggest SHFL could effectively inhibit viruses in flavivirus family.

### 2.4 Restricting the Gag/Gag-Pol ratio of HIV

HIV is a member of the Lentivirus genus of the Retroviridae family, and possesses two uniform copies of single-stranded RNA. HIV (including HIV-1 and HIV-2) contains three representative structural genes: gag, pol, and env. The formation of the Gag and Pol proteins requires the cleavage of a large 160 kDa precursor molecule by a protease (Fanales-Belasio et al., 2010). HIV-1 Gag carries a−1PRF signal near its open reading frame (ORF) terminal and impels the occurrence of −1PRF, resulting in the formation of Gag–Pol complex. Precise regulation of the ratio of Gag to Gag–Pol is necessary for HIV assembly, genome packaging, and maturation (Wang et al., 2019).

Except for directly restricting the ratio of Gag to Gag–Pol, SHFL can interact with the ribosomal proteins, such as uL5, eS31, and eRF3, and the −1PRF signal-carrying RNA. This shows that SHFL, which also has a limiting effect on HIV-1 replication, can inhibit the −1PRF of HIV-1. The region for antiviral activity including the 164th–199th amino acid of SHFL, is required for HIV-1 inhibition and the binding activity of SHFL (Wang et al., 2019; Napthine et al., 2021). These results suggest that the zinc-finger domain of SHFL might produce a key effect in HIV infection by binding −1PRF and regulating the ratio of Gag/ Gag–Pol.

### 2.5 Inhibiting −1PRF transcription of JEV

JEV, a positive-sense, single-stranded RNA virus, is a zoonotic mosquito-borne flavivirus that can cause a series of infectious diseases of the central nervous system (Yun and Lee, 2013). The whole genome of JEV is 11 kb, including three structural proteins C, M, and E and nonstructural proteins (from NS1 to NS5). The JEV polyprotein can be cleaved by viral and host proteases (Sharma et al., 2021). The NS3 protein has a peculiar structure and multiple enzyme activities, which is also regarded as the constituent of the JEV RNA replicase and participates in the replication of JEV (Yu et al., 2021). Similar to Gag protein of HIV, production of NS1 protein depends on −1PRF (Chen et al., 1997).

SHFL can inhibit NS3 protein via the lysosome-dependent pathway and−1PRF by downregulating the expression of the NS1 protein and further decrease the JEV titer. However, the mRNA expressing level of NS3 or NS1 is not affected. Moreover, the zinc-finger domain of SHFL is still important in JEV inhibition (Yu et al., 2021). Together with Wang’s study (Wang et al., 2019), it seems that inhibit −1PRF progress is one of the main anti-viral mechanisms of SHFL.

### 2.6 Inducing degradation of PEDV N protein

PEDV is a single-stranded, positive-sense RNA virus, belonging to the genus Alphacoronavirus, family *Coronaviridae*, and the cause of porcine epidemic diarrhea (Sun et al., 2012). PEDV genome encodes four structural proteins (spike protein [S], envelope protein [E], membrane protein [M], and nucleocapsid protein [N]), 16 nonstructural proteins (nsp1–nsp16), and one accessory protein (ORF3) (Kocherhans et al., 2001). The N proteins play crucial roles in PEDV replication and transcription (Lin et al., 2022).

PEDV infection induces the early growth response gene 1 (EGR1) expression in the host cells, which can up-regulate SHFL expression at both protein and mRNA levels by directly binding to the SHFL promoter. The highly expressed SHFL can directly interact with the N protein and stimulate its degradation depending on the ubiquitin-proteasome and autolysosome pathways, and further inhibit PEDV replication. The anti- PEDV activity of SHFL are possibly related to the E3 ubiquitin ligase membrane-associated RING-CH8 (Wang et al., 2021).

### 2.7 Downregulating 3D protein of EV-A71 by SHFL

Human enterovirus A71 (EV-A71) belongs to Picornavirus family, which is a non-enveloped and single-strand RNA virus. The RNA encodes a polyprotein with three regions (P1, P2 and P3), which contains four structural proteins (VP1–VP4) and seven nonstructural proteins (2A, 2B, 2C, 3A, 3B, 3C, and 3D). The 3D protein of EV-A71 is an RNA-dependent polymerase determined the viral genome synthesis (Yuan et al., 2018). EV-A71 infection could lead to the hand–foot and mouth disease (HFMD), encephalitis, and aseptic meningitis worldwide (Huang and Shih, 2014; Kinobe et al., 2022).

Recently, EV-A71 is reported to facilitate the expression of SHFL (Tan et al., 2023). SHFL interacts with and downregulates viral 3D protein by the K48 ubiquitin–protease pathway. Then, EV-A71 propagation was inhibited. Tan et al. (2023) also found that the zinc-finger domain and 36 amino acids (164–199) were crucial for SHFL to bind with 3D protein.

Above all, SHFL showed wide-spectrum against RNA virus. It seems that some common anti-viral mechanisms are identified when SHFL inhibit RNA viruses, such as binding to RdRp protein of virus, suppressing −1PRF. In addition, SHFL might be induced not only by IFN but also by other factors, i.e., EGR1 (Woodson and Kehn-Hall, 2022).

## 3 The inhibition function of SHFL to DNA virus

### 3.1 Inhibiting expression of crucial genes in KSHV

KSHV, also known as human herpesvirus-8, is a Rhadinovirus of the gamma herpesvirus subfamily. During infection, the genome of this linearized double-stranded DNA virus circularizes to form an episome and remains present in the nucleus (Veettil et al., 2014). The life cycle of KSHV is divided into the two phases: the latency and the lytic phase. These two phases ensure KSHV could continuously survive in the host and infect to new individual (Broussard and Damania, 2020). The shutoff and exonuclease (SOX) protein of KSHV can degrade mRNAs in the host cells through targeting the special motif off most host mRNAs (Rodriguez et al., 2019).

SHFL contains a long stem-loop structure in the 3’UTR semblable with SOX resistance element, which could protect host mRNAs from destruction. In addition, SHFL can inhibit the KSHV replication and mobilize immune factors’ activities of host (Rodriguez et al., 2019, 2022). However, the anti-KSHV mechanisms of SHFL is not clear. We prefer to that SHFL inhibit KSHV through other pathways differed from that of anti-RNA virus. Although SHFL shows the activity to inhibit the viral titer of herpes simplex virus and adenovirus, but the anti-viral mechanisms are not studied (Suzuki et al., 2016).

### 3.2 Genetic association between the *SHFL* gene and hepatitis B virus (HBV)

HBV is an enveloped DNA virus belonging to the *Hepadnaviridae* family and causes advanced liver disease and hepatocellular carcinoma (Li et al., 2022). In our previous studies, the association between HBV infection in the Yunnan population and the genetic polymorphisms in the interleukin 28B, IFN lambda 4, myxovirus resistance (Mx)A, and MxB genes has been determined (Zhang et al., 2014; Zheng et al., 2022). Although there is no direct evidence that SHFL inhibits HBV proliferation, the genetic polymorphisms of the SHFL gene have been related to the biochemical indices of HBV patients in Yunnan. Inversely, the single nucleotide polymorphisms with different genotypes of SHFL might affect its expression by changing the association of transcriptional factors (Liu et al., 2023). This result displayed the relationship between HBV infection/ disease progression of HBV patients and the genetic characteristics of SHFL. However, whether SHFL could inhibit HBV replication need further study.

## 4 Future perspectives of SHFL

SHFL has garnered the attention of researchers owing to its particular structure and diverse virus-inhibiting function. In this review, we aimed to provide a comprehensive understanding of the antiviral functions of SHFL and the virus-associated specific molecules involved in inhibition processes. However, the following questions remain unanswered and need further study:

- Because most anti-viral studies of SHFL focus on the zinc-finger domain and 164th–199th amino acid, the function of other domains of SHFL are needed further investigate. The coiled-coil domain showed important roles in anti-viral activity of some ISGs, such as TRIM5α (Song, 2009). Thus, we speculate that the coiled-coil domain of SHFL might play important roles.
- Both human SHFL and mouse Shfl gene showed wide-spectrum antiviral activities. Thus, this gene might be against zoonosis viruses.
- Until now, although SHFL could inhibit both DNA viruses and RNA viruses, the species of DNA virus are rare. In addition, the anti-viral mechanisms are distinct among different viruses (Table 1), but it is still unclear in some virus infection. Thus, more studies should be further performed in various viruses.

**Table 1:** Summary of the antiviral function of SHFL.

Viruses	Mainly processes of inhibition	References
DENV	Suppress DENV mRNA Interact with host immune factors Act with DENV replication complex	Suzuki et al. (2016) and Balinsky et al. (2017)
ZIKV	Restrict ZIKV RNA expression Degrade NS3 protein	Wu et al. (2020)
HCV	Decrease NS5A expression Destroy membranous web formation Combine with HCV replicase complex	Kinast et al. (2020)
HIV-1	Inhibit -1PRF Regulate Gag–Pol ratio	Wang et al. (2019) and Napthine et al. (2021)
JEV	Inhibit -1PRF Degrade NS3 protein	Yu et al. (2021)
PEDV	Degrade NS3 protein	Wang et al. (2021)
EV-A71	Interact with 3D protein Degrade NS3 protein	Tan et al. (2023)
KSHV	Suppress viral gene expression Protect host mRNAs from destruction	Rodriguez et al. (2019) and Rodriguez et al. (2022)

In conclusion, we summarized the functional characteristics of SHFL. SHFL showed widely anti-viral spectrum in both DNA and RNA viruses. The studies suggest that the functional structure of SHFL is the zinc-finger domain, especial the 164th–199th amino acids. The anti-viral mechanisms of SHFL focus on inhibiting -1PRF, degrading RdRp of viruses, decreasing viral RNA expressing level. It is worth much to study the SHFL in future.

## Author contributions

XW: Writing – original draft. A-MZ: Conceptualization, Writing – review & editing.

## Footnotes

^1^www.ncbi.nlm.nih.gov
